# Immediate effects of photobiomodulation on maximum lip pressure

**DOI:** 10.1590/2317-1782/20212021024

**Published:** 2022-01-05

**Authors:** Vanessa Mouffron, Renata Maria Moreira Moraes Furlan, Andréa Rodrigues Motta

**Affiliations:** 1 Programa de Pós-graduação em Ciências Fonoaudiológicas, Universidade Federal de Minas Gerais – UFMG - Belo Horizonte (MG), Brasil.; 2 Departamento de Fonoaudiologia, Faculdade de Medicina, Universidade Federal de Minas Gerais – UFMG - Belo Horizonte (MG), Brasil.

**Keywords:** Low-level Light Therapy, Lip, Muscle Strenght, Speech, Language and Hearing Sciences, Myofunctional Therapy

## Abstract

**Purpose:**

To verify the immediate effects of different doses of photobiomodulation on maximum lip pressure.

**Methods:**

Experimental, randomized and triple-blind study. The sample consisted of 23 women and 17 men, age between 18 and 33 years old (average 23.18 years old, SD=2.1), distributed in four groups: CG (control group), G1, G4 and G7. The maximum pressure was assessed with the Iowa Oral Performance Instrument (IOPI). The bulb was placed between the lips and the participants were instructed to press it as strong as possible. Infrared LASER (808 nm), manufactured by DMC, Therapy EC model, 100 mW of power output, was applied. The doses tested were 1 J (G1), 4 J (G4) and 7 J (G7), applied at six points of the orbicularis oris muscle. In the CG there was no intervention. The evaluation procedures were repeated after the LASER application. The results were analyzed with a significance level of 95%.

**Results:**

The maximum lip pressure increased significantly only in the group irradiated with 7 J.

**Conclusion:**

Low level LASER therapy with 7 J dose promoted changes in the performance of the orbicularis oris muscle in the maximum pressure task.

## INTRODUCTION

Photobiomodulation therapy is a form of light therapy based on non-ionizing forms of light sources, including LASER, LEDs and/or broadband lights, non-visible and infrared spectrum. It is a non-thermal process that causes photophysical and photochemical events on different biological scales^(1)^. The technique is non-invasive and painless, and has no side effects, thus representing little risk to the patient^(2,3)^.

The main mechanism of action of this resource consists of the photochemical effect, through which light energy is absorbed by organelles named as chromophores, present especially in mitochondria, and transformed into chemical energy during the cellular respiration process. Through enzymatic reactions, LASER stimulates an increase in adenosine triphosphate (ATP) synthesis, an essential substance for the adequate cell functioning^(4)^.

Muscle performance can be defined as the capacity of the muscle to produce work, which, among other factors, can be affected by morphological characteristics, metabolic, cardiovascular, respiratory, cognitive, and emotional functions^(5)^. Considering the great energy demand during a muscle activity, both in contraction and relaxation processes and maintenance of body tonus^(6)^, the photobiomodulation effects on this tissue have triggered interest in different areas. In this context, studies have revealed the efficiency of applying therapeutic LASER or LED to improve muscle performance^(7)^, delayed fatigue^(8)^, and reduce muscle damage after high-demand activities^(9)^.

Among the orofacial muscles, the orbicularis oris – which constitutes the lips – is highly important for the performance of functions such as speech, chewing, swallowing, breathing, and facial mimicry. Alterations in this muscle tension can trigger problems like slurred speech and lip seal difficulties, which can comprise chewing and swallowing, and even cause alterations in the dental arch, since the proper lip tension opposes to the pressure resistance exerted by the tongue on the teeth. Thus, the treatment of patients with alterations in the stomatognathic functions should encompass muscle training for the orbicularis oris^(10)^. In Speech Therapy, interesting results have emerged from associating LASER with myofunctional training^(11,12)^ whose effects and related dosimetry parameters on such structure can contribute significantly to scientific evidence on the topic.

Considering this, this study was performed aiming to verify the immediate effects of different doses of Photobiomodulation using low-level laser on maximum lip pressure.

## METHODS

This is a randomized, triple-blind clinical trial (RBR-563rmd), approved by the Research Ethics Committee of the Federal University of Minas Gerais – protocol number CAAE 83652117,6,0000,5149.

Non-probabilistic sample composed of 40 individuals: 23 (57.5%) women and 17 (42.5%) men, with a minimum age of 18 years old and a maximum age of 33 years old (average of 23.18 years, SD=2.1), who accepted the invitation to participate in the research. All participants signed an Informed Consent Form (ICF).

The participants were recruited through posters and personal invitations made in the institution. The following inclusion criteria were established: individuals aged between 18 and 35 years old, without myorelaxant and/or anti-inflammatory medication, no prior speech therapy, and no contraindications to phototherapy^(13)^, which, according to the manufacturer’s manual and specific literature, are photosensitivity, pregnancy, glaucoma, undiagnosed lesion on or near the area to be irradiated, infection at the application site, history of cancer, use of a pacemaker or other electronic implant. The only exclusion criterion was the non-execution of all proposed tasks.

Each participant was instructed to remain seated in a chair maintaining upright posture, 90° flexion of hips, knees, and ankles, guided by the Frankfurt plane. The procedures of assessment and application of low-level laser were performed by different researchers. It is worth highlighting that the experimental group received only one LASER application.

**Figure 1:**
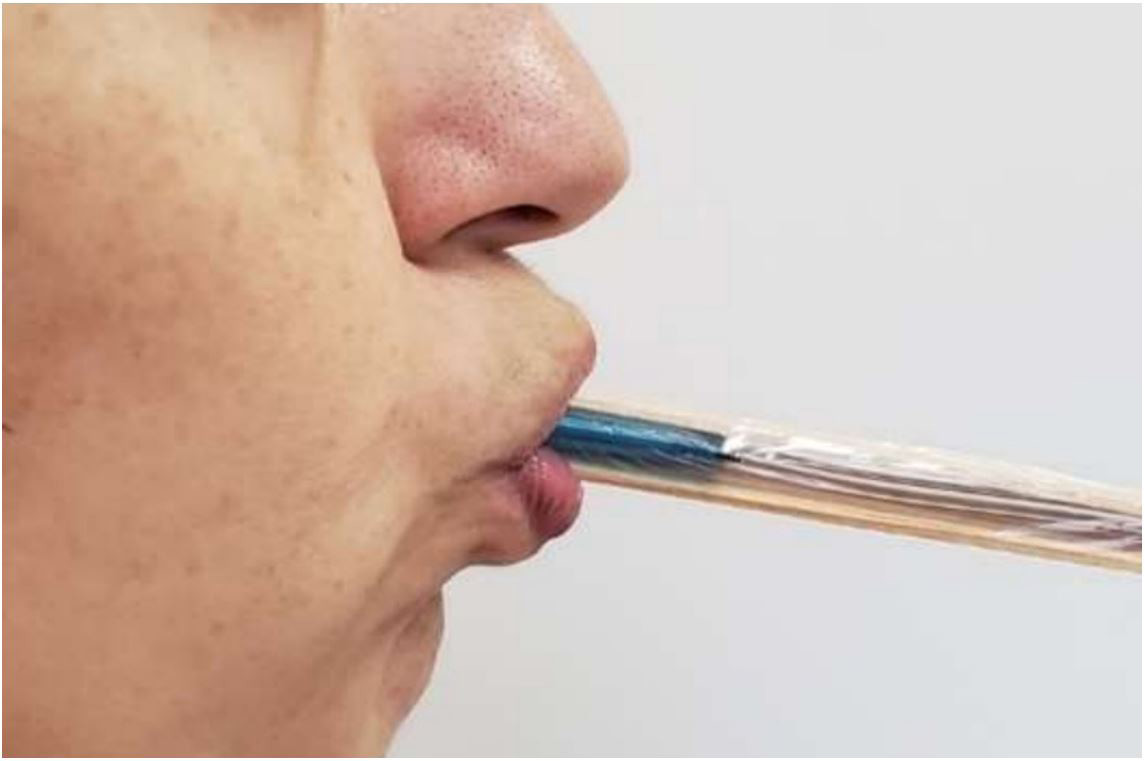
Assessment of lip strength through IOPI

The Iowa Oral Performance Instrument (IOPI) was applied to obtain the maximum lip pressure values. The IOPI device consists of an air bulb connected to a pressure transducer, which allows measuring the maximum pressure and muscle resistance. The air balloon has 3.5 cm length, 1.0 cm diameter and is connected to a plastic tube of 11.5 cm. As the bulb is pressed, the device captures the generated change in pressure, providing values in kPa that are visualized on the LCD screen of the device. In our experiments, the bulb was positioned between two wooden spatulas and the set was wrapped with plastic film, as in Figure 1. The choice to use spatulas was based on a previous study using the same methodology, which described that such configuration distributed the pressure exerted by the lips uniformly on the entire bulb surface, thus providing an accurate reading of the pressure^(14)^.

The participants were instructed to press the bulb with as much strength as possible, with lip gripping movement, for two seconds – following the procedures in previous studies on IOPI^(15,16)^. It was performed three repetitions with a thirty-second interval between them^(17)^ considering the maximum peak value among all repetitions as the maximum pressure.

After the initial assessment, the photobiomodulation was performed using a LASER device manufactured by DMC, model Therapy EC, with 100 mW of power output and spot size of 0.028 cm^2^. For this study, it was used infrared wavelength (808 nm) and doses of 1 J, 4 J, and 7 J.

Due to the lack of studies addressing the topic, we decided to investigate the action of three different irradiation doses to establish the greatest influence on lip muscle behavior. The choice of dosimetry parameters was based on other studies that specifically used low-level LASER as resource to improve muscle activity^(18,19)^.

The dose of 1 J was chosen for being close to the dose applied at the only study reported aimed at assessing the effects of LASER on the performance of a facial muscle without correlation to analgesic effect. The referred study performed an irradiation of 0.8 J on the masseter muscle and found an increase in its electric activity, but without effects on either strength or fatigue^(18)^. Due to both the power and model of the equipment used in this study, the authors chose to apply the dose of 1 J for the irradiation time to be automatic, thus eliminating possible bias in the application.

Doses of 4 J and 7 J were chosen due to the fact that they are commonly found in studies on the effect of low-level laser on muscle performance, according to recent bibliographical review^(19)^. Infrared wavelength has also been indicated as preferable by several authors^(19)^, which supported the choice of use.

**Figure 2:**
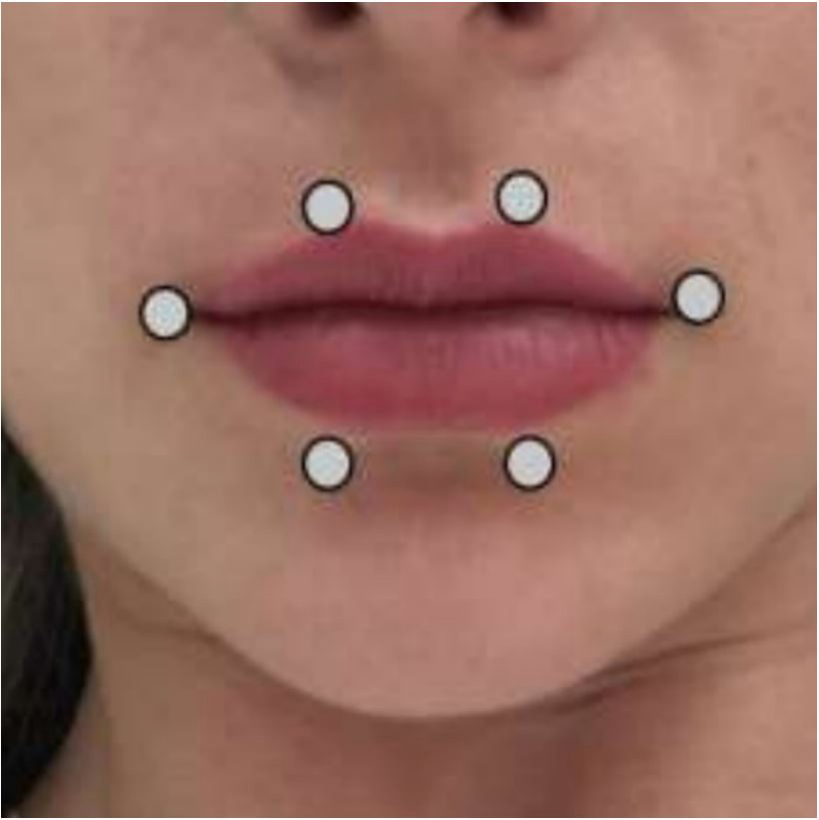
Points of laser application.

The application was carried out in a timely manner, with contact at six equidistant points on the orbicularis oris muscle: near the right and left commissures (for being a motor point region), two points at the upper part, and two other points at the lower part (Figure 2).

The participants were randomly and blindly distributed in the following groups: control group (GC) (n=10), G1(n=10), G4 (n=10), and G7 (n=10), with the following doses applied to each group:

- Control group: without LASER application;
- G1: one dose of 1 J per point, energy density of 35 J/cm^2^ per point, application time of 10 seconds per point – total amount of 6 J on the muscle;
- G4: 4 J per point, energy density of 140 J/cm^2^, application time of 40 seconds per point – total amount of 24 J on the muscle;
- G7: 7 J per point, energy density of 245 J/cm^2^, application time of 70 seconds per point – total amount of 42 J on the muscle.

Following the manufacturer recommendations and meeting the safety standards established by ANVISA for low-level laser equipment, the researcher responsible for the application and the participants wore goggles throughout the entire procedure.

After applying the LASER, the assessment procedures for maximum lip pressure were repeated. All assessments were performed by one researcher, who had not been informed about into which group the participant had been allocated. The control group was subjected to a two-minute interval between the measures aiming to provide the necessary rest for the musculature to recover^(17,20)^.

For data analysis, the files were renamed so that the researcher responsible for the analysis did not know the participant's group or the moment of the assessment (pre- or post- LASER application).

The description of the gender category variable was based on frequency measures, while the comparative analysis between the variables of gender and group was performed through a chi-square test for multiple comparisons. The continuous variables (age and result of each task) were described based on the measures of central tendency and dispersion, whereas the normality distribution of the variables was assessed through a Shapiro-Wilk test, which indicated normal distribution. Thus, we chose to apply non-parametric tests to the data analysis. The comparative analysis between the variables of age and group was carried out through a Kruskal Wallis test and the results of the pre- and post- LASER tests were compared through Wilcoxon test.

## RESULTS

The results indicate no statistically significant difference when comparing the groups regarding both gender (p= 0.975) and age (p=0.353), therefor, the groups had homogeneous values for such variables.

Table 1 presents the comparison of the results from the pre- and post-LASER IOPI per group. Maximum lip pressure was higher only in the group irradiated with 7 J.

**Table 1:** Measures of lip pressure in kPa pre- and post-laser per group

**Group**	**Control**		**1 J**		**4 J**		**7 J**	
	**Pre**	**Post**	**Pre**	**Post**	**Pre**	**Post**	**Pre**	**Post**
Average	11.80	11.70	12.90	12.90	11.50	12.20	10.60	11.90
Median	11.50	12.50	12.50	13.00	11.00	13.00	10.50	11.50
SD	3.77	3.27	3.60	4.70	2.12	2.15	2.63	2.88
Minimum	6.00	6.00	7.00	6.00	9.00	7.00	7.00	8.00
Maximum	20.00	15.00	18.00	20.00	15.00	14.00	14.00	16.00
p value^*^	1.000		1.000		0.250		**0.013**	

* Wilcoxon Test

Caption: J = joule; SD = standard deviation

## DISCUSSION

The study found that photobiomodulation with low-level laser promoted changes in the performance of the orbicularis oris muscle, assessed here through maximum lip pressure.

The effects of low-level laser on the muscle tissue have been drawing the interest of research groups, and some studies have already revealed positive results on the performance and recovery of lesions^(7,21)^. Nonetheless, most of these studies analyzed muscle groups in upper or lower limbs, whereas no studies were found addressing the effects of this resource on the performance of the orbicularis oris muscle.

Authors decided to apply the infrared wavelength tested in three different doses. However, it is known that the choice of wavelength is directly related to the depth of the target tissue. Considering the muscle thickness in question, we believe that red wavelength can be an efficient resource as well, which can be further explored in additional studies. The simultaneous application of the two wavelengths is also worth investigating since recent studies using equipment with LASER and LED have highlighted such association as an interesting alternative to muscle performance^(22)^.

The groups in this study were homogeneous regarding gender and age, which represents a relevant information since lip strength has direct influence of these two variables^(23)^. The use of the IOPI was based in previous studies in which maximum lip pressure was used as assessment parameter for the muscle activity^(14,24,25)^.

Our findings include a significant increase in maximum lip pressure at the dose of 7 J per point, which is in line with other studies that also reached positive responses for muscle performance at the same dose on quadriceps and rectus femoris muscles by assessing the number of repetitions, electromyographic fatigue, and lactate levels^(26-28)^.

The group irradiated with 4 J did not show statistically significant difference in the maximum pressure levels, which corroborates an earlier study that did not achieve significant responses in athletes’ muscle performance after application on rectus femoris, assessed through specific test^(29)^. However, another study that applied the same dose per point observed a significant increase in the number of repetitions, as well as electromyographic fatigue delay after irradiation in lower limbs^(30)^. Although, it is worth considering that the former study used a total dose of 20 J, while the latter involved an irradiation on rectus femoris, vastus medialis, vastus lateralis at a total dose of 60 J. Such difference in the final dose can be among the factors that may have interfered with the diverging results.

This study did not find significant results for the dose of 1 J, which is aligned with the current reports in literature, in which the dose did not promote an increase in masseter muscle strength^(18)^.

Despite not significant, the group irradiated with 4 J showed an increase in the average maximum pressure, which did not occur either in the control group or the group irradiated with 1 J.

Such an improvement trend in the results at higher doses corroborates other studies reporting that the most satisfactory total doses for small muscles range the interval from 20 J to 60 J^(22)^. Therefore, in this study, the total dose of 6 J exerted no effects on the orbicular muscle, whereas a slight increase occurred at the total dose of 24 J and a significant result was found for the total dose of 42 J.

Still on the divergence of results in studies that used the same dose, it is important to consider that the studies differ, among other aspects, by the total irradiated dose, moment of application, number of points, and irradiated muscles, thus demonstrating that dosimetry photobiomodulation parameters are not restricted to wavelength or energy applied per point. This is also a major obstacle to compare studies, as well as to reproductivity, especially due to the large variety of equipment available for research groups.

Since photobiomodulation can interfere directly with these biochemical mechanisms of contraction, contributing especially to the ATP input, its action on mitochondria is believed to have greater influence on resistance exercises, which was not covered in this study. For this reason, it is suggested that additional studies analyze the laser therapy effects on fatigue, for which electromyography is a good resource.

The positive results for maximum lip pressure are believed to demonstrate that the low-level laser irradiation can influence muscle activity. This is an innovative study since the literature has no other reports on the assessment of photobiomodulation effects on the performance of the orbicularis oris muscle. Nonetheless, it is worth regarding the exploratory nature of this research, involving a small sample, thus imposing the need for further studies to identify other dosimetry parameters that could promote a greater performance of this muscle, as well as the clinical applicability of such findings.

Finally, authors emphasizes that much is yet to be elucidated on the photobiomodulation mechanisms of action on muscle performance; additionally, this study focused on discussing papers that used exclusively LASER as therapeutic resource. Authors acknowledge the increasing importance of LEDs in speech therapy and muscle performance. The studies reported in the literature use very different methodologies, which are often not very thorough, in addition to several dosimetry parameters, different equipment models and nomenclatures. These factors hamper the comparison of results and the scientific proof of the resource efficiency since some positive findings were based on questionable methodologies.

## CONCLUSION

Low-level laser at the dose of 7 J promoted changes in the performance of orbicularis oris muscle in maximum pressure task.
