# Supplementary material for: Immediate effects of photobiomodulation on maximum lip pressure
Source: Codas. 2022 Jan 5;34(2):e20210024. doi: 10.1590/2317-1782/20212021024 (PMC9769423; doi:10.1590/2317-1782/20212021024)
Supplement: Translation

# Translation: Efeitos imediatos da fotobiomodulação sobre a pressão máxima dos lábios

**Authors:** Vanessa Mouffron, Renata Maria Moreira Moraes Furlan, Andréa Rodrigues Motta

**Affiliations:** 1 Programa de Pós-graduação em Ciências Fonoaudiológicas, Universidade Federal de Minas Gerais – UFMG - Belo Horizonte (MG), Brasil.; 2 Departamento de Fonoaudiologia, Faculdade de Medicina, Universidade Federal de Minas Gerais – UFMG - Belo Horizonte (MG), Brasil.

**Keywords:** Terapia com Luz de Baixa Intensidade, Lábio, Força Muscular, Fonoaudiologia, Terapia Miofuncional

## Abstract

**Objetivo:**

Verificar os efeitos imediatos da fotobiomodulação sobre a pressão máxima dos lábios para diferentes doses.

**Método:**

Estudo experimental, randomizado e triplo cego. A amostra foi composta por 23 mulheres e 17 homens com idade entre 18 e 33 anos (média 23,18 anos, DP=2,1), divididos em quatro grupos: GC (grupo controle), G1, G4 e G7. A pressão máxima foi avaliada com o Iowa Oral Performance Instrument (IOPI). O bulbo foi posicionado entre os lábios e os participantes foram orientados a pressioná-lo com a maior força possível. Aplicou-se o LASER infravermelho (808 nm) da marca DMC, modelo Therapy EC, 100 mW de potência. As doses testadas foram 1 J (G1), 4 J (G4) e 7 J (G7) aplicadas em seis pontos do músculo orbicular da boca. No GC não houve intervenção. Após a aplicação do LASER, foram repetidos os procedimentos de avaliação. Os resultados foram analisados com nível de significância de 95%.

**Resultados:**

A pressão máxima de lábios aumentou significativamente apenas no grupo irradiado com 7 joules.

**Conclusão:**

O LASER de baixa intensidade na dose de 7 J promoveu mudanças no desempenho do músculo orbicular da boca em tarefa de pressão máxima.

## INTRODUÇÃO

A terapia por fotobiomodulação é uma forma de terapia luminosa que utiliza formas não ionizantes de fontes de luz, incluindo LASER, LEDs e/ou luzes de banda larga, no espectro visível e infravermelho. É um processo não térmico que provoca eventos fotofísicos e fotoquímicos em várias escalas biológicas^(1)^. Trata-se de uma técnica não invasiva, indolor, com baixo risco para o paciente e sem efeitos colaterais^(2,3)^.

O principal mecanismo de ação desse recurso consiste no chamado efeito fotoquímico. De acordo com essa teoria, a energia luminosa é absorvida por organelas denominadas cromóforos, presentes principalmente nas mitocôndrias, e transformada em energia química durante o processo de respiração celular. Por meio de reações enzimáticas, o LASER estimula o aumento da síntese de adenosina trifosfato (ATP), substância essencial para o adequado funcionamento das células^(4)^.

O desempenho muscular pode ser definido como a capacidade do músculo de produzir trabalho e, dentre outros fatores, pode ser afetado pelas características morfológicas, por funções metabólicas, cardiovasculares, respiratória, cognitiva e emocional^(5)^. Considerando que há grande demanda energética durante uma atividade muscular, tanto para os processos de contração como também de relaxamento e manutenção do tônus corporal^(6)^, os efeitos da fotobiomodulação sobre esse tecido tem despertado o interesse de diferentes áreas, com pesquisas revelando eficácia do uso de LASER ou LED terapêutico para melhora do desempenho muscular^(7)^, atraso na fadiga^(8)^ e na redução de dano muscular após atividades de alta demanda^(9)^.

Dentre os músculos orofaciais, o orbicular da boca – que constitui os lábios - é de grande importância no desempenho de funções como a fala, mastigação, deglutição, respiração e mímica facial. Alterações na tensão dessa musculatura podem desencadear problemas como imprecisão articulatória e dificuldades no vedamento labial, as quais podem comprometer a mastigação, a deglutição e até mesmo acarretar alterações na arcada dentária, uma vez que a tensão adequada dos lábios opõe resistência à pressão que a língua exerce sobre os dentes. Por esses motivos, o treinamento muscular do orbicular da boca pode ser um dos aspectos a serem trabalhados na terapia de pacientes com alterações das funções estomatognáticas^(10)^. Na Fonoaudiologia, a associação do LASER ao treino miofuncional tem trazido resultados interessantes^(11,12)^ e acredita-se que entender os efeitos desse recurso e seus parâmetros dosimétricos sobre essa estrutura será de grande contribuição para o crescimento das evidências científicas acerca do assunto.

Diante disso, o objetivo deste trabalho foi verificar os efeitos imediatos da fotobiomodulação com LASER de baixa intensidade sobre a pressão máxima dos lábios para diferentes doses.

## MÉTODOS

Trata-se de um ensaio clínico randomizado e triplo cego (RBR-563rmd), aprovado pelo Comitê de Ética em Pesquisa da Universidade Federal de Minas Gerais, sob parecer número CAAE 83652117.6.0000.5149.

A amostra, não probabilística, foi composta por 40 indivíduos, sendo 23 (57,5%) do sexo feminino e 17 (42,5%) do sexo masculino, com idade mínima de 18, máxima de 33 e média de 23,18 anos (DP=2,1) que aceitaram o convite para participar da pesquisa. Todos os participantes assinaram o Termo de Consentimento Livre e Esclarecido (TCLE).

Os participantes foram recrutados por meio de cartazes e convites pessoais realizados na própria instituição e foram estabelecidos como critérios de inclusão: ter idade entre 18 e 35 anos, não fazer uso de medicação miorrelaxante e/ou anti-inflamatória, não ter realizado fonoterapia prévia e não apresentar contraindicações para fototerapia, conforme manual dos fabricantes e literatura específica^(13)^. São essas: fotossensibilidade, gravidez, glaucoma, lesão sem diagnóstico sobre a área a ser irradiada ou próxima a ela, infecção no local da aplicação, histórico de câncer, uso de marcapasso ou outro implante eletrônico. Adotou-se como critério de exclusão a não execução de todas as tarefas propostas.

Cada participante foi orientado a permanecer sentado em uma cadeira mantendo postura ereta, flexão de 90° de quadril, de joelhos e de tornozelos, guiados pelo Plano de Frankfurt. Os procedimentos de avaliação e de aplicação do LASER de baixa intensidade foram realizados por diferentes pesquisadoras. Ressalta-se, ainda, que o grupo experimental recebeu apenas uma aplicação do LASER.

Para obtenção dos valores de pressão máxima dos lábios utilizou-se o *Iowa Oral Performance Instrument (IOPI).* O IOPI é um aparelho constituído por um bulbo de ar ligado a um transdutor de pressão, que permite mensurar a pressão máxima e a resistência muscular. O balão de ar apresenta 3,5 cm de comprimento, 1,0 cm de diâmetro e conecta-se a um tubo plástico de 11,5 cm. À medida que esse bulbo é pressionado, o aparelho capta a mudança de pressão gerada, fornecendo valores em kPa que podem ser visualizados na tela de LCD do próprio aparelho. Nesta pesquisa, o bulbo foi colocado entre duas espátulas de madeira e o conjunto foi envolto com filme plástico conforme Figura 1. Optou-se pelo uso das espátulas, pois um estudo anterior, no qual a mesma metodologia foi utilizada, descreveu que essa configuração distribuiu a pressão exercida pelos lábios de maneira uniforme em toda a superfície do bulbo, fornecendo uma leitura precisa da pressão^(14)^.

**Figura 1:** Avaliação da força labial utilizando-se o IOPI

Os participantes foram solicitados a pressionar o bulbo com o máximo de força possível, com movimento de preensão dos lábios, durante 2 segundos, conforme estudos anteriores também realizados com o IOPI^(15,16)^. Foram realizadas três repetições, com intervalo de trinta segundos entre elas^(17)^ e o valor do pico máximo dentre todas as repetições foi considerado a pressão máxima.

Após a avaliação inicial, realizou-se a aplicação do LASER de baixa intensidade. O equipamento utilizado foi da marca DMC, modelo Therapy EC, com 100 mW de potência e *spot* de saída com área de 0,028 cm^2^. Para esse estudo optou-se pelo comprimento de onda infravermelho (808 nm) e as doses de 1 J, 4 J e 7 J.

Devido à escassez de estudos sobre o tema, nesta pesquisa optou-se por investigar a ação de três diferentes doses de irradiação, a fim de estabelecer aquela que poderia apresentar maior influência no comportamento do lábio. A escolha de alguns parâmetros dosimétricos baseou-se em pesquisas anteriores que utilizaram especificamente o LASER de baixa intensidade como recurso para promover melhora da atividade muscular^(18,19)^.

A dose de 1 J foi escolhida por ser próxima à utilizada no único trabalho encontrado com o objetivo de avaliar os efeitos do LASER no desempenho de um músculo da face sem correlação com o efeito analgésico. No estudo em questão, foi feita a irradiação de 0,8 J sobre o músculo masseter e observou-se aumento na atividade elétrica desse músculo, porém sem efeitos sobre força e fadiga^(18)^. Devido à potência e modelo do equipamento utilizado nesse estudo, as autoras optaram por utilizar a dose de 1 J para que o tempo de irradiação fosse automático, eliminando, assim, possível viés relacionado à aplicação.

As doses de 4 J e 7 J, por sua vez, foram selecionadas por serem as mais encontradas em artigos que também investigaram a ação do LASER de baixa intensidade no desempenho muscular, conforme revisão bibliográfica recente^(19)^. O comprimento de onda infravermelho também foi apontado como o preferido por diversos autores^(19)^ e, por esse motivo, utilizado nesta pesquisa.

A aplicação foi feita de modo pontual, com contato, em seis pontos equidistantes no músculo orbicular da boca: próximo às comissuras direita e esquerda (por ser região dos pontos motores), dois pontos na porção superior e dois pontos na porção inferior (Figura 2).

**Figura 2:** Pontos de aplicação do LASER

Os participantes foram alocados, de maneira randomizada e cega, em um dos seguintes grupos: grupo controle (GC) (n=10), G1(n=10), G4 (n=10) e G7 (n=10) e as doses aplicadas em cada um deles foram as seguintes:

- Grupo controle: não houve aplicação do LASER;
- G1: aplicou-se dose de 1 J por ponto, densidade de energia de 35 J/cm^2^ em cada ponto, tempo de aplicação de 10 segundos por ponto, totalizando 6 J no músculo;
- G4: aplicou-se 4 J por ponto, densidade de energia de 140 J/cm^2^, tempo de aplicação de 40 segundos por ponto, totalizando 24 J no músculo;
- G7: aplicou-se 7 J por ponto, com densidade de energia de 245 J/cm^2^, tempo de aplicação de 70 segundos por ponto e dose total no músculo de 42 J.

Seguindo-se recomendações do fabricante e atendendo às normas de segurança estabelecidas pela ANVISA para equipamentos de LASER de baixa intensidade, a pesquisadora responsável pela aplicação e os participantes usaram óculos de proteção durante todo o procedimento.

Após a aplicação do LASER, foram repetidos os procedimentos de avaliação da pressão máxima dos lábios. Todas as avaliações foram realizadas por uma das pesquisadoras, que não sabia a qual grupo o participante pertencia. No grupo controle, o intervalo entre as medições foi de dois minutos, a fim de proporcionar o repouso necessário para restabelecimento da musculatura^(17,20)^.

Para análise dos dados, os arquivos foram renomeados de modo que a pesquisadora responsável pela análise não soubesse o grupo do participante e nem o momento da avaliação (pré ou pós aplicação do LASER).

A descrição da variável categórica sexo foi realizada por meio de medidas de frequência e a análise comparativa entre as variáveis sexo e grupo foi realizada por meio do teste qui-quadrado de comparações múltiplas. A descrição das variáveis contínuas (idade e resultado de cada tarefa) foi realizada por meio de medidas de tendência central e de dispersão e a distribuição da normalidade das variáveis foi avaliada por meio do teste Shapiro-Wilk, o qual indicou que essas variáveis não apresentaram distribuição normal. Assim, optou-se pela utilização de testes não paramétricos para a análise dos dados. A análise comparativa entre as variáveis idade e grupo foi realizada por meio do teste Kruskal Wallis e a comparação entre os resultados dos exames pré e pós LASER foi realizada por meio do teste Wilcoxon.

## RESULTADOS

Os resultados indicam que não houve diferença com relevância estatística ao comparar os grupos em relação ao sexo (p= 0,975) e idade (p=0,353), ou seja, os grupos foram homogêneos em relação a essas variáveis.

A Tabela 1 apresenta a comparação dos resultados obtidos por meio do IOPI antes e após o LASER em cada grupo. A pressão máxima de lábios apresentou aumento apenas no grupo submetido à irradiação com 7 J.

**Tabela 1:** Medidas da pressão labial em kPa antes e após o LASER em cada grupo

**Grupo**	**Controle**		**1 J**		**4 J**		**7 J**	
	**Pré**	**Pós**	**Pré**	**Pós**	**Pré**	**Pós**	**Pré**	**Pós**
Média	11,80	11,70	12,90	12,90	11,50	12,20	10,60	11,90
Mediana	11,50	12,50	12,50	13,00	11,00	13,00	10,50	11,50
DP	3,77	3,27	3,60	4,70	2,12	2,15	2,63	2,88
Mínimo	6,00	6,00	7,00	6,00	9,00	7,00	7,00	8,00
Máximo	20,00	15,00	18,00	20,00	15,00	14,00	14,00	16,00
valor de p*	1,000		1,000		0,250		**0,013**	

* Teste de Wilcoxon

Legenda: J = joule; DP = desvio-padrão.

## DISCUSSÃO

Observou-se, nesse estudo, que a fotobiomodulação com LASER de baixa intensidade promoveu mudanças no desempenho do músculo orbicular da boca, aqui avaliado por meio da pressão máxima de lábios.

Os efeitos do LASER de baixa intensidade no tecido muscular vêm despertando o interesse de grupos de pesquisas e estudos anteriores já revelaram resultados positivos sobre o desempenho e recuperação de lesões^(7,21)^. No entanto, grande parte desses trabalhos foram realizados com grupos musculares dos membros superiores ou inferiores, não tendo sido encontrado nenhum trabalho que investigasse os efeitos desse recurso no desempenho do músculo orbicular da boca.

Nesse estudo, optou-se pelo uso do comprimento de onda infravermelho, testado em três diferentes doses. No entanto, sabe-se que a escolha do comprimento de onda tem relação direta com a profundidade do tecido alvo. Devido à espessura do músculo em questão, acredita-se que o comprimento de onda vermelho também possa ser um recurso eficaz, o que pode ser investigado em pesquisas futuras. A aplicação simultânea dos dois comprimentos de onda também merece ser pesquisada, uma vez que pesquisas recentes, utilizando equipamentos com LASER e LED têm mostrado essa associação como uma opção interessante para desempenho muscular^(22)^.

Os grupos participantes desse estudo foram homogêneos em relação ao sexo e à idade, dado esse relevante uma vez que a força labial sofre influência direta dessas duas variáveis^(23)^. A escolha pelo IOPI baseou-se em estudos anteriores, nos quais a pressão máxima de lábios foi utilizada como parâmetro de avaliação da atividade muscular^(14,24,25)^.

Em relação aos achados, observou-se o aumento significativo da pressão máxima dos lábios para a dose de 7 J por ponto, o que está de acordo com outros estudos que também obtiveram respostas positivas para desempenho muscular com essa dose aplicada nos músculos quadríceps e reto femoral quando avaliados o número de repetições, a fadiga eletromiográfica e níveis de lactato^(26-28)^.

No grupo irradiado com 4 J não houve diferença estatisticamente significativa nos níveis de pressão máxima, o que está de acordo com estudo anterior que também não obteve respostas significativas na performance muscular de atletas após aplicação no reto-femoral, avaliada por meio do teste específico^(29)^. No entanto, um outro estudo que utilizou essa mesma dose por ponto observou, sim, aumento significativo do número de repetições e atraso da fadiga eletromiográfica após irradiação em membros inferiores^(30)^. Há de se considerar, porém, que no primeiro estudo a dose total utilizada foi de 20 J, enquanto no segundo trabalho a irradiação foi feita sobre o reto-femoral, vasto medial, vasto lateral com dose total de 60 J. Tal diferença na dose final pode ser um dos fatores que tenham interferido nessa divergência de resultados.

Nesta pesquisa não foram encontrados resultados significativos para a dose de 1 J, achado esse que está de acordo com a literatura existente, na qual essa dose não promoveu aumento de força do músculo masseter^(18)^.

Apesar de não significativo, no grupo irradiado com 4 J houve um aumento da média da pressão máxima, o que não foi observado no grupo controle e no grupo irradiado com 1 J.

Essa tendência de melhora dos resultados com o aumento das doses é coerente com o apresentado por outros autores, os quais referem que as melhores doses totais, para músculos pequenos, estão no intervalo de 20 J a 60 J^(22)^. Dessa forma, neste trabalho a dose total de 6 J não surtiu efeitos para o músculo orbicular da boca, observou-se um discreto aumento com a dose total de 24 J e o resultado foi significativo para dose total de 42 J.

Ainda sobre a divergência de resultados em estudos que utilizaram a mesma dose, é importante considerar que os artigos se diferenciam, dentre outros aspectos, pela dose total irradiada, pelo momento de aplicação, número de pontos e pelos músculos irradiados, o que demonstra que os parâmetros dosimétricos em fotobiomodulação não se restringem ao comprimento de onda e à energia utilizada por ponto. Tal fato é, ainda, um grande dificultador para a comparação entre os estudos, assim como para sua reprodutibilidade, sobretudo pela grande variedade de equipamentos disponíveis para os grupos de pesquisa.

Uma vez que a fotobiomodulação pode interferir diretamente nesses mecanismos bioquímicos de contração, contribuindo sobretudo para o aporte de ATP, acredita-se que sua ação nas mitocôndrias possa ter maior influência sobre exercícios de resistência, o que não foi investigado neste estudo. Por isso, sugere-se que pesquisas futuras analisem os efeitos da laserterapia sobre a fadiga, sendo a eletromiografia um bom recurso para tal.

Acredita-se que os resultados positivos para pressão máxima do lábio demonstram que a irradiação com o LASER de baixa intensidade é capaz de influenciar na atividade da musculatura. Trata-se de um trabalho inovador, uma vez que não foram encontrados na literatura estudos anteriores que tenham avaliado os efeitos da fotobiomodulação sobre o desempenho do músculo orbicular da boca. No entanto, há de se considerar que essa é uma pesquisa exploratória, com amostra pequena e que outros trabalhos precisam ser conduzidos a fim de identificar quais outros parâmetros dosimétricos podem promover um maior desempenho desse músculo e as aplicabilidades clínicas desses achados.

Por fim, ressalta-se que há muito o que ser esclarecido sobre os mecanismos de ação da fotobiomodulação no desempenho muscular e que este trabalho teve como foco principal a discussão de artigos que utilizaram apenas o LASER como recurso terapêutico. As autoras reconhecem a crescente importância dos LEDs na terapêutica fonoaudiológica e também no desempenho muscular. Os trabalhos encontrados na literatura possuem metodologias muito diferentes e, por vezes, pouco criteriosas. Somado a isso, são encontrados diversos parâmetros dosimétricos, diferentes modelos de equipamentos e diferentes nomenclaturas. Tais fatos dificultam a comparação de resultados e também a comprovação científica da eficácia do recurso, uma vez que alguns achados positivos apresentam metodologias duvidosas.

## CONCLUSÃO

O LASER de baixa intensidade na dose de 7 J promoveu mudanças no desempenho do músculo orbicular da boca em tarefa de pressão máxima.
